# Heterogeneous zinc/catechol-derived resin microsphere-functionalized composite hydrogels with antibacterial and anti-inflammatory activities promote bacterial-infected wound healing

**DOI:** 10.1093/rb/rbaf081

**Published:** 2025-08-09

**Authors:** Lianyi Qu, Anle Yang, Yulei Shi, Jianglong Liu, Xueyan Li, Bohan Mao, Xiaoran Li, Fang Zhou, Yingjun Xu

**Affiliations:** College of Textiles & Clothing, Institute of Functional Textiles and Advanced Materials, Qingdao 266071, China; College of Textiles & Clothing, Institute of Functional Textiles and Advanced Materials, Qingdao 266071, China; College of Textiles & Clothing, Institute of Functional Textiles and Advanced Materials, Qingdao 266071, China; College of Textiles & Clothing, Institute of Functional Textiles and Advanced Materials, Qingdao 266071, China; College of Textiles & Clothing, Institute of Functional Textiles and Advanced Materials, Qingdao 266071, China; College of Textiles & Clothing, Institute of Functional Textiles and Advanced Materials, Qingdao 266071, China; Innovation Center for Textile Science and Technology, College of Textiles, Donghua University, Shanghai 201620, China; College of Textiles & Clothing, Institute of Functional Textiles and Advanced Materials, Qingdao 266071, China; College of Textiles & Clothing, Institute of Functional Textiles and Advanced Materials, Qingdao 266071, China

**Keywords:** composite hydrogel, microsphere, antibacterial activity, macrophage polarization, wound healing

## Abstract

Bacterial infection in the injured skin may threaten the wound repair and skin regeneration owing to aggravated inflammation. The multifunctional dressings with persistent antibacterial activity and improved anti-inflammatory capability are urgently required. Herein, a type of heterogeneous zinc/catechol-derived resin microspheres (Zn/CFRs) composed of zinc ions (Zn^2+^) and zinc oxide (ZnO) nanoparticles was developed to impart the methacrylamide chitosan (CSMA)-oxidized hyaluronic acid (OHA) hydrogel with a persistent Zn^2+^ release behavior. The Zn/CFRs synthesized via a one-step hydrothermal method exhibited a Zn^2+^-enriched surface and internal ZnO nanoparticles. Owing to the unique microstructure of the microspheres, the Zn/CFRs-functionalized hydrogel (CH-ZnCFR) was able to rapidly release Zn^2+^ in the initial phase and sustain the release of Zn^2+^ for 14 days. Importantly, CH-ZnCFR exhibited excellent anti-inflammatory property by facilitating the macrophage polarization, and also effectively inhibited the growth of *Staphylococcus aureus* and *Escherichia coli*. In addition, CH-ZnCFR showed excellent self-healing and tissue adhesion properties, and great cytocompatibility by improving fibroblast migration behavior *in vitro*. Moreover, CH-ZnCFR demonstrated outstanding therapeutic effects in a murine model of *S. aureus*-infected wounds, including effectively inhibiting bacterial growth, reducing inflammation, increasing the number of M2-type macrophages and facilitating collagen deposition, angiogenesis and tissue regeneration. Therefore, this Zn/CFRs-functionalized composite hydrogel represents a promising strategy for bacterial-infected wound healing and regeneration.

## Graphical abstract

**Figure:**
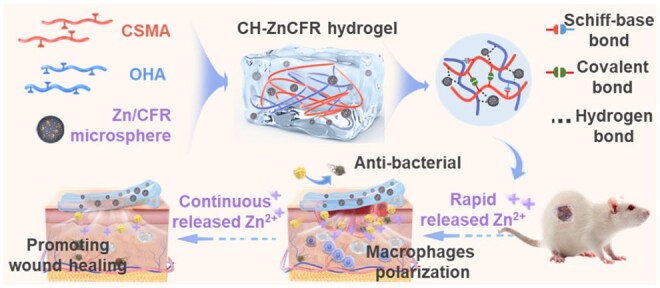

## Introduction

Chronic wounds, such as pressure, venous and diabetic ulcers, typically possess persistent bacterial infections, high levels of inflammation and poor angiogenesis [1, 2]. Bacterial invasion and infections exacerbate skin damage and result in heavy financial burdens on healthcare institutions [3, 4]. To date, various wound dressings, such as hydrogels, sponges and fibrous membranes, have been developed to protect against bacterial infections and promote chronic wound repair [5–9]. Among them, hydrogels containing extracellular matrix-like structures, superior moisture retention and biocompatibilities, have been developed as promising dressing materials [9–12]. Ideal hydrogel dressings should not only possess proper adhesive and self-healing properties to prevent accidental detachment, but also have effective antibacterial and anti-inflammatory capabilities to overcome the infected-wound microenvironment [3, 7, 9]. Although numerous hydrogel dressings have been developed, the development of dressings with persistent antibacterial activity and improved anti-inflammatory capability that meet various requirements for chronic wound repair is still challenging.

Recently, metal-based hydrogels have been widely developed to repair chronic skin defects [13–18]. Zinc (Zn), an essential trace element in our body, plays important roles in human physiology and pathology. Besides, Zn-based biomaterials could promote fibroblast proliferation and inhibit bacterial reproduction [15–17]. In particular, zinc oxide (ZnO) nanoparticle-loaded scaffolds were able to continuously release zinc ions (Zn^2+^) from wounds, demonstrating great potential for wound repair [16, 19, 20]. Whereas, the clinical application of ZnO-based wound dressings is limited due to the stable crystal structure of ZnO nanoparticles, which greatly hinders the release of Zn^2+^ and decreases the antibacterial efficiency [20, 21]. Simultaneously, many researchers found that Zn^2+^-loaded composite biomaterials were able to rapidly release Zn^2+^ in a short period of time [15, 21, 22]. Ju *et al.* prepared Zn^2+^ cross-linked alginate hydrogel microspheres, which exert effective antibacterial and anti-inflammatory effects, remodeling wound microenvironments [22]. Recently, metal–phenolic networks or hydrogels have garnered significant attention due to their excellent antibacterial, antioxidant and anti-inflammatory capacities [21–24]. Metal–phenolic networks are usually formed utilizing the coordination between metal ions and phenolic ligands (such as tannic acid, catechol, *et al.*). However, metal–catechol networks are prone to degradation under external or internal environmental stimuli (such as temperature and pH), resulting in unstable and excessively rapid release of metal ions in the body, leading to excessive accumulation of metal ions in a short period of time and causing damage to tissues and human body [23–25]. Considering the sophisticated cascade involved in chronic wound repair, an ideal Zn-based hydrogel dressing (incorporating either ZnO nanoparticles or metal–phenolic networks) should demonstrate phase-specific Zn^2+^ release kinetics—rapid release of antimicrobial Zn^2+^ during the initial inflammatory stage, followed by sustained release during subsequent proliferative and tissue remodeling stages.

Catechol-derived resins, which are polymers rich in phenolic hydroxyl groups, have been demonstrated to exhibit low cytotoxicity and excellent adhesive properties [23–25]. Catechol-derived resins are effective platforms for the fabrication of composite biomaterials used in tissue engineering fields [26–28]. Metal ions, such as silver and ferric ions, with strong oxidation, can react with catechol groups rapidly to form silver nanoparticles and Fe_3_O_4_ nanoparticles [29, 30]. And silver/catechol-derived resin microspheres have been developed previously *via* a facile hydrothermal redox reaction [31], which demonstrated positive effects on skin wound healing [32]. In particular, Zn^2+^ are nonoxidative metal ions, and Zn-based catechol-derived resin spheres are difficult to prepare by such methods [33]. As far as we know, few reports have been published on the formation of Zn/catechol-derived resin sphere or hydrogel dressings.

**Figure 1.**
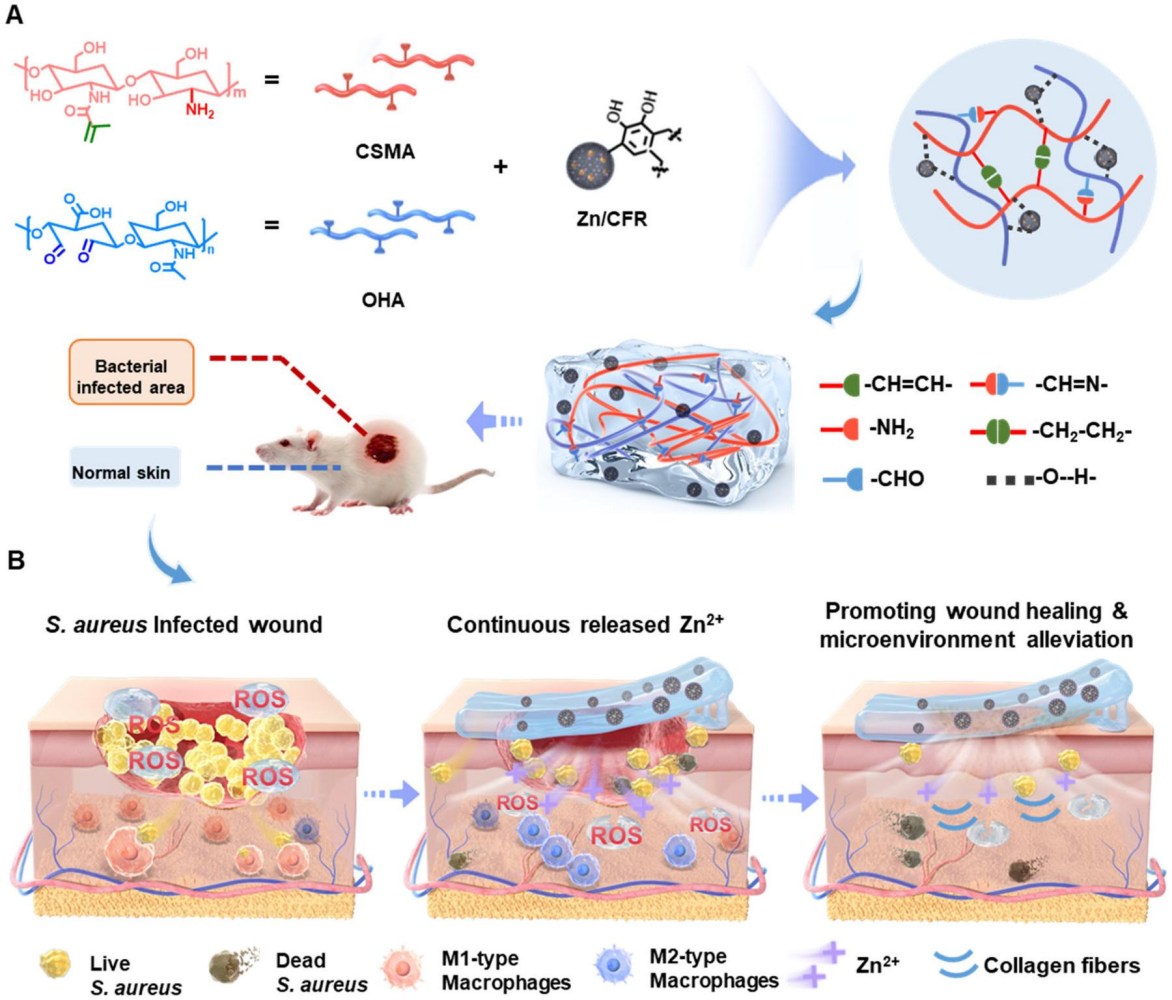
Schematic illustration of the fabrication of a Zn/CFR-loaded hydrogel (CH-ZnCFR) for skin tissue regeneration applications. (**A**) Synthesis of Zn/CFR microspheres and preparation of the microsphere-loaded hydrogel. (**B**) Antibacterial, immunomodulatory and re-epithelialization effects of the hydrogel.

Herein, a type of heterogeneous Zn-based catechol-derived resin microspheres (denoted as Zn/CFRs) was designed to impart composite hydrogels persistent antibacterial activity and improved anti-inflammatory capability during wound healing process (Figure 1). The Zn/CFRs were fabricated via a one-pot hydrothermal method, and exhibited a Zn^2+^-enriched surface and internal ZnO nanoparticles. We propose that Zn/CFRs with unique microstructures can persist release of Zn^2+^ release to overcome the infected wound conditions. Chitosan-based hydrogels are being actively studied for wound repair due to their good biocompatibility, biodegradability, low immunogenicity and sustained bioactive drug delivery [32, 34–38]. Hyaluronic acid (HA) is an endogenous substance inherently found within the human body, widely distributed in the extracellular matrix and exhibits exceptional biocompatibility without raising immunogenicity concerns [32]. Thus, the Zn/CFRs-functionalized composite hydrogels (CH-ZnCFR) were formed by dual cross-linking strategy including photo-crosslinking of methacrylamide chitosan (CSMA) and Schiff-base reactions between the amino groups of CSMA and aldehyde groups of oxidized hyaluronic acid (OHA), which constructed the hydrogel network with great biocompatibility. CH-ZnCFR exhibited great self-healing, tissue adhesion properties and enhanced fibroblast migration behavior. Besides, the CH-ZnCFR can notably suppress bacteria growth and facilitate macrophage polarization. The regenerative capabilities of the CH-ZnCFR composite hydrogel to reduce inflammation, increase collagen deposition and promote wound healing were validated by expanding our investigation to a chronic infected wound healing model.

## Materials and methods

### Materials

ZnCl_2_ (AR. 99%), hexamethylenetetramine (HMT, 99.5%) and catechol (AR, 99%) were acquired from Kelong Chemical Corporation (Chengdu, China). HA, chitosan (85% deacetylation), sodium periodate (99.5%), methacrylic anhydride (94%), 2,2-diphenyl-1-picrylhydrazyl (DPPH), polyvinyl pyrrolidone (PVP) and other chemical reagents were obtained from Shanghai Macklin Biochemical Co., Ltd, China. The 1000 iFluor™ 488 phalloidin, 4′, 6-diamidino-2-phenylindole (DAPI) and other cell culture reagents were provided by Meilun Biotechnology Co., Ltd (Dalian, China). Mouse fibroblasts cells (L929) and RAW 264.7 were purchased from iCell Bioscience Inc, Shanghai, China. Harris hematoxylin (H&E) and Masson’s trichrome (M&T) stain kits, anti-CD86, anti-CD206, anti-CD31, anti-*α*-SMA antibody and ELISA kits were purchased from Boster Biological Technology Co., Ltd, China.

### Preparation and characterization of the Zn/CFR microspheres

The synthesis of Zn/CFRs was achieved via a hydrothermal approach, utilizing HMT, catechol and ZnCl_2_ as precursors. Briefly, 0.15, 0.3 and/or 0.45 mmol ZnCl_2_ was introduced into 400 mL deionized water. The mixture containing 1.5 mmol HMT, 3 mmol catechol and various amounts of PVP (0.04, 0.2 and 0.4 g) was subsequently slowly added into the previously mentioned solution. This mixture was stirred at room temperature until a clear and uniform solution was obtained, which was then moved into a Teflon-sealed stainless-steel autoclave and heated in an oven at 120, 160 and/or 200°C for 3, 6 and/or 9 h, respectively. Following the reaction, precipitates were centrifuged and washed with deionized water. The obtained microspheres were ultimately dried at 80°C.

The morphological structure of Zn/CFRs was determined by scanning electronic microscope (SEM) and transmission electron microscopy (TEM). The elemental compositions of Zn/CFRs were tested by X-ray powder diffraction (XRD) and X-ray photoelectron spectroscopy (XPS). The chemical structure of Zn/CFRs was assessed by Fourier transform infrared spectroscopy (FTIR) and solid-state ^13^C nuclear magnetic resonance spectroscopy (^13^C NMR, Bruker, Germany). The antibacterial property, Zn^2+^ release behavior, cell toxicity, dispersive stability and adhesion force of the Zn/CFRs were also characterized. All the specific procedures of the aforementioned tests were described in the Supporting Information.

### Preparation and characterization of the CH-ZnCFR hydrogels

Oxidized hyaluronic acid (OHA) and methacrylamide chitosan (CSMA) were synthesized following previous studies [38], with detailed procedures provided in the Supporting Information. Then, 2.0 mg of Zn/CFRs were added to a 2 mL aqueous solution of 2.5 wt% CSMA. Meanwhile, 0.1 wt% of 2959 photoinitiator was added to a 1 mL aqueous solution of 5.0 wt% OHA. The obtained solutions were mixed and stirred well. The resulting mixture loading Zn/CFRs was exposed to UV radiation for 20 s. The obtained hydrogel was designated as CH-ZnCFR. The hydrogel prepared without the inclusion of Zn/CFRs was referred to as CH.

Zn^2+^ release amounts from the hydrogels were tested by inductively coupled plasma atomic emission spectrometry (ICP-MS, Agilent 7800, USA). The detailed procedure of the swelling ratios, water retention ratios, degradation property, rheological, adhesion and antioxidant characterization of the CH-ZnCFR hydrogels are also presented in the Supporting Information. Given the disparities in size and morphology between nanoparticles and hydrogels, the antibacterial properties of different hydrogels against *Escherichia coli* and *Staphylococcus aureus* were assessed by the colony count method [9, 16]. The detailed procedures are shown in the Supporting Information.

### *In vitro* cell viability

Cytocompatibility of the CH-ZnCFR hydrogel was determined through extract analysis, adhering to ISO 10993 standards [39]. Briefly, lyophilized hydrogel disks (1 cm in diameter) were soaked in culture medium for 24 h. L929 cells were subsequently seeded into 96-well plates (1 × 10^4^ cells/well) and exposed to the hydrogel extracts for 1 and 3 days. Cell viability was evaluated via a CCK-8 assay at 1 and 3 days after cell seeding. The migration capability of fibroblasts was assessed with a scratch wound assay [25]. L929 cells were seeded in 24-well plates (1 × 10^4^ cells/well) and cultured for 24 h. A scratch was then created on the cell layer with a 20 μL pipette tip. After the floating cells were removed, the cells were treated with the hydrogel extracts. The cells were photographed with a light microscope after 24 h of incubation, and the degree of cell migration was calculated. The hemocompatibility of the CH-ZnCFR hydrogels was characterized, and the specific procedures are described in the Supporting Information.

### Expression of inflammation-related factors

Following an overnight culture of RAW 264.7 cells, the lipopolysaccharide (LPS, 50 ng/mL) was used as the inflammatory stimulus to activate macrophages. RAW 264.7 cells were then rinsed with PBS following the removal of the medium containing LPS and incubated with the hydrogel extracts for a day. Afterwards, the cells were cultured in a new serum-free culture medium for 24 h. The inflammatory factor level of *TNF-α*, *IL-10* and *IL-6* was measured via ELISA kits.

### *In vivo* infected wound healing assessment

Male Kunming (KM) mice (weighing about 40 g) were provided by Huafukang Biotechnology Co. Ltd (Beijing, China), under the animal license SCXK (Jing) 2023-0003. All animal procedures adhered to the guidelines outlined in the National Research Council Guidelines for the Care and Use of Laboratory Animals and were approved by the Ethics Committee of Qingdao University School of Medicine (QDU-AEC-2022380). Briefly, Male KM mice were randomly assigned to four groups (*n *= 5), namely, Control, 3M Tegaderm™, CH and CH-ZnCFR groups. Following anesthesia and depilation, a full-thickness skin wound (1 cm diameter) was created on the dorsal skin of each mouse via surgical scissors. Meanwhile, the wound sites were treated with 200 µl of *S. aureus* (1 × 10^8^ CFU/mL) applied drop-by-drop. Following 24 h, the hydrogels were placed over the full-thickness wounds. The wound area was monitored and photographed on day 3, 7 and 14 after wound creation. The mice were sacrificed on day 3 and tissues from the infected wound were isolated and put into sterile centrifuge tube carrying 2 mL LB broth. The diluted LB broth, carrying bacteria from infected wounds, was placed on agar plates for 18 h at 37°C. The antibacterial effects of the hydrogels *in vivo* were assessed by the colony count method.

For histological analysis, the tissues were collected on day 7 and 14, and fixed in 4% paraformaldehyde. After the tissue sections were embedded and section-processed, H&E and M&T staining were conducted [40]. Additionally, immunofluorescence staining was performed using specific antibodies against CD86 (1:100) and CD206 (1:100). Antibodies against CD31 (1:100) and *α-SMA* (1:100) were used to assess angiogenesis. The wound size and fluorescence intensity of the staining markers were quantitatively analysed via ImageJ software. The *in vivo* hemostatic effects of the CH-ZnCFR hydrogels were characterized. The specific procedures are described in the Supporting Information.

### Statistical analysis

The quantitative data were analysed *via* the one-way ANOVA test. The experimental data were obtained from at least three independent replicates. *P *< 0.05 was considered to indicate a statistically significant difference (*P < 0.05, **P < 0.01 and ***P < 0.001).

## Results and discussion

### Synthesis and characterization of Zn/CFRs

Zn/CFRs were synthesized via a hydrothermal approach. The reactions in hydrothermal systems are highly complex and involve multiple factors, which leads to a variety of diverse and sometimes contentious reaction mechanisms. However, on the basis of reports of the synthesis of phenolic resin microspheres and hybrid colloidal particles, the key parameters influencing the growth kinetics of these systems include the molar ratio of precursors, temperature and surfactant [41, 42]. Therefore, we systematically investigated the effects of the molar ratio of catechol to Zn^2+^, reaction time, reaction temperature and the dosage of PVP on Zn/CFRs growth and optimized the microspheres synthesis conditions according to the established criteria.

**Figure 2.**
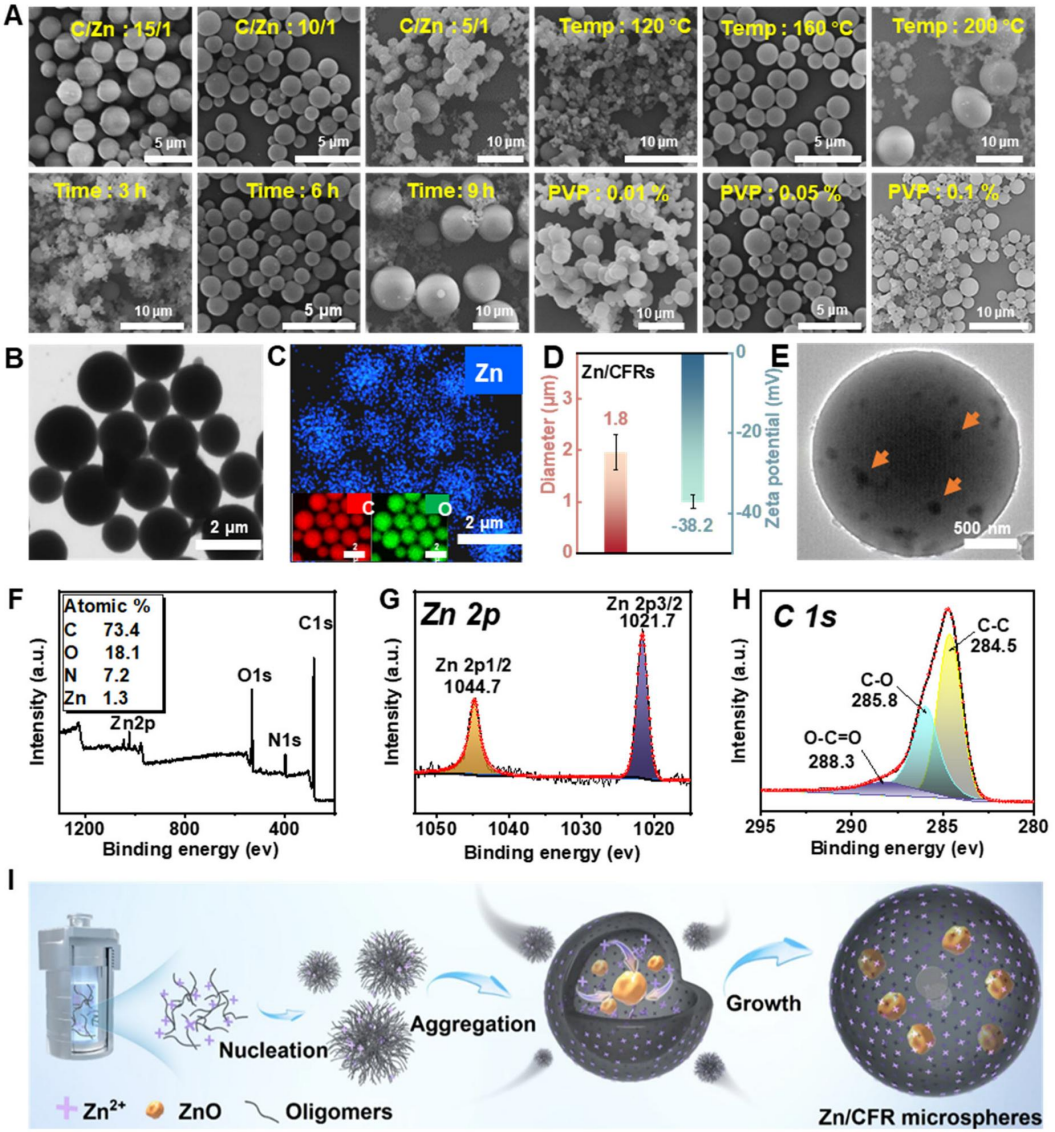
Morphological and chemical structure analysis of Zn/CFRs. (**A**) SEM images of Zn/CFRs prepared under different synthesis conditions. (**B**) SEM images, (**C**) elemental mapping images, (**D**) diameter and zeta potential, (**E**) TEM images, (**F**) full XPS survey spectrum of the Zn/CFRs, high-resolution spectra of (**G**) Zn 2p and (**H**) C 1 s. (**I**) Schematic illustration of the mechanism of the Zn/CFR formation.

SEM images of the products with different synthesis conditions are present in Figure 2A. The morphology of the microspheres varied under different synthesis conditions. Specifically, when the Zn^2+^ concentration was low, microsphere formation remained unchanged, but as the concentration increased, the product changed to an irregular structure. At lower reaction temperatures and shorter reaction times, the product consists of small microspheres and irregular particles, whereas higher temperatures and longer reaction times result in larger microspheres alongside assorted particles. Additionally, a low amount of surfactant also influences the formation of microspheres. Finally, Zn/CFRs with a regular morphology can be successfully synthesized by maintaining the molar ratio of catechol to Zn^2+^ at 10:1, conducting the reaction at a temperature of 160°C for a duration of 6 h and incorporating PVP at a dosage of 0.05%. SEM and EDX images of the Zn/CFRs (Figure 2B and C) further confirmed that the Zn/CFRs appeared as uniformly distributed monodisperse microspheres, with C, O and Zn elements evenly distributed on the surface. The diameter and zeta potential of the Zn/CFRs (Figure 2D) were 1.8 μm and −38.2 mV, respectively, indicating good dispersibility of the Zn/CFRs in aqueous solution. TEM images of the Zn/CFRs (Figure 2E) revealed that some irregular particles were randomly distributed inside the microspheres. Furthermore, the inner surface of the Zn/CFRs was further investigated via TEM-EDS coupled with a focused ion beam (FIB) (Supplementary Figures S1 and S2), which also revealed that Zn was evenly distributed inside the microspheres. On the basis of these observations, it can be concluded that the Zn/CFRs had smooth surfaces with evenly distributed Zn^2+^ and contained some irregular particles internally.

Figure 2F presents the XPS spectrum of Zn/CFRs, showing C, O, N and Zn elements, with the Zn content reaching 1.3%. The presence of N was attributed to ${\text{NH}}_{4}^{+}$, which was one of the byproducts of the experimental reaction. The Zn 2p high-resolution spectrum of the Zn/CFRs (Figure 2G) displayed a double band for Zn 2p_1/2_ and Zn 2p_3/2_, with two strong peaks at 1044.7 and 1021.7 eV and a doublet splitting of around 23.0 eV, which indicates that the Zn in the microspheres existed in its oxidized state [43, 44]. Figure 2H shows the C 1 s spectrum of the Zn/CFRs, revealing three peaks at 288.6, 285.6 and 284.5 eV, corresponding to the O–C=O, C–O and C–C bonds of the CFR, respectively. Additionally, the chemical structure of Zn/CFRs was also determined via FTIR and ^13^C NMR (Supplementary Figure S3), confirming that the resin framework structure of the Zn/CFRs [45, 46]. XRD and electron paramagnetic resonance (EPR) were further conducted to explore the state of the Zn. As shown in Supplementary Figures S4 and S5, the XRD spectrum did not exhibit distinct characteristic peaks, whereas the EPR spectrum revealed clear peaks associated with hydroxyl radicals. The generation of reactive oxygen species under UV light is a unique feature of ZnO, revealing the presence of ZnO NPs within the Zn/CFRs [47, 48]. The absence of distinct peaks in the XRD spectrum might be due to the ZnO NPs being embedded within the Zn/CFRs and their quantity falling below the detection limit. Therefore, we propose that Zn/CFRs are multicomponent heterogeneous structures characterized by surface-complexed Zn^2+^ and randomly distributed ZnO NPs within the internal matrix.

Through the above analysis, it is inferred that the construction of heterogeneous Zn/CFRs involves two main reactions occurring in the system (Figure 2I). (1) Catechol first forms a complex with Zn^2+^, and then reacts with derived under alkaline conditions to produce Zn^2+^-containing oligomers. These oligomers self-assemble into clusters, which eventually form spherical structures due to surface tension and cross-linking effects. (2) In addition, the chemical environments inside and outside the microspheres gradually differed. Specifically, the Zn^2+^ on the surface of the microspheres were impacted by solvents and ammonia, remaining in a complex state. In contrast, the Zn^2+^ within the microsphere, devoid of these influences, gradually dissociated from the complex and subsequently underwent conversion into ZnO NPs under such thermal conditions. Therefore, we believe that the formation of Zn/CFRs with heterogeneous structures is determined by the regulation of synthesis conditions, with the following two points. First, during the formation of microspheres, significant differences in the microchemical environment between the inside and outside of the microspheres were generated. Second, control of the synthesis conditions especially the reaction time and reaction temperature, ensured that the formation of the microspheres was well coordinated with the orderly growth of the ZnO NPs. Otherwise, the incomplete formation of the resin microspheres or the disordered overgrowth of ZnO NPs within the microspheres resulted in the failure of the synthesis of Zn/CFRs with targeted structures.

### Antibacterial and dispersion stability evaluation of Zn/CFRs

**Figure 3.**
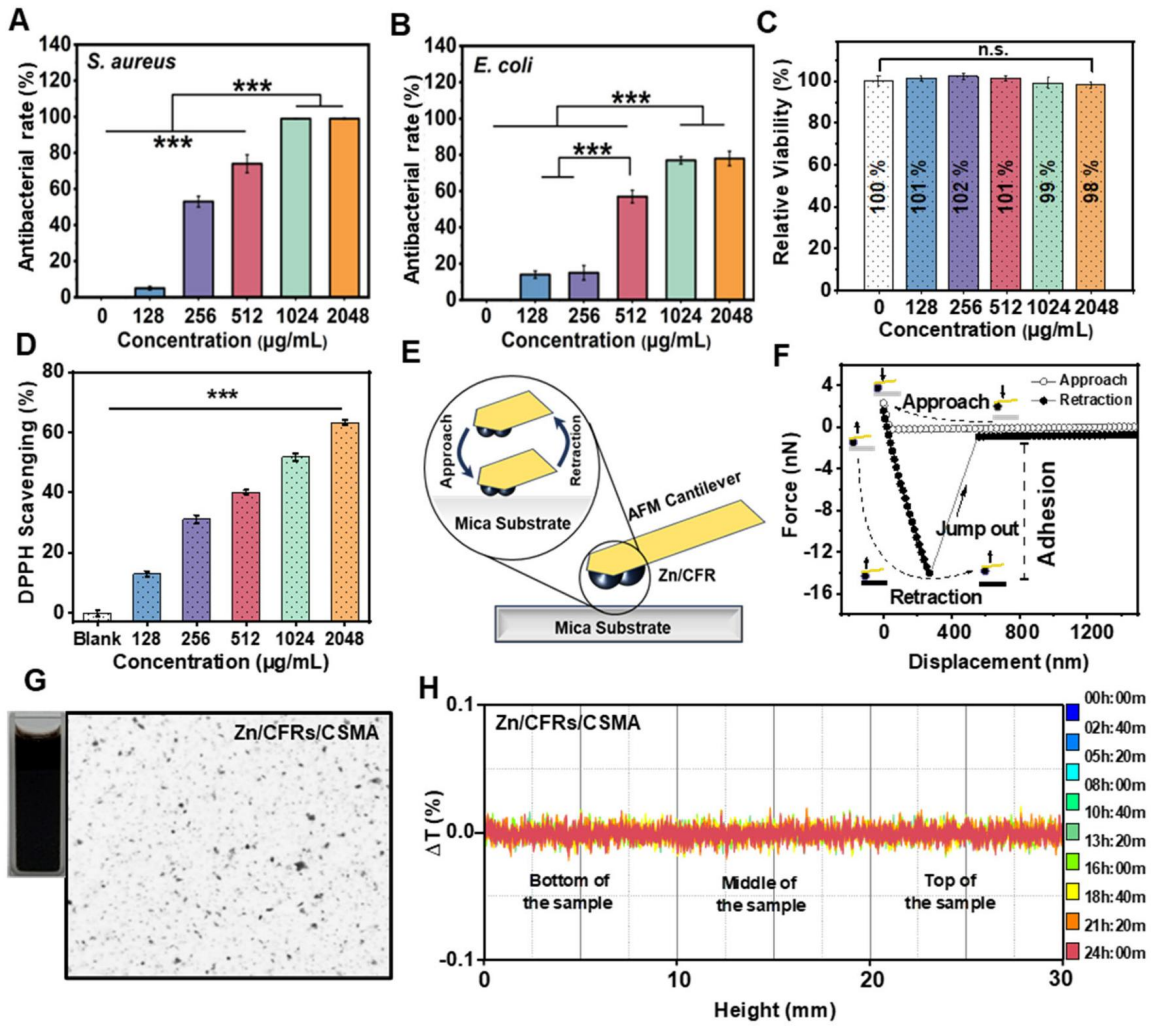
Antibacterial, cytotoxic, adhesion, dispersion and stability evaluation of the Zn/CFRs. Antibacterial rates of the Zn/CFRs against (**A**) *Staphylococcus aureus* and (**B**) *Escherichia coli*. (**C**) Cell viability and (**D**) DPPH scavenging ability of the Zn/CFRs. Schematic description of (**E**) AFM force measurements and (**F**) force–distance curves. (**G**) Digital photographs and optical microscope images of a CSMA solution containing Zn/CFRs. (**H**) Δ*T* versus the height of the Zn/CFR CSMA solution over 24 h.

The antibacterial property of Zn/CFRs in the suspension was evaluated employing *S. aureus* and *E. coli* as representative strains. As shown in Figure 3A and B, the lowest concentrations of Zn/CFRs that can greatly inhibit the *S. aureus* and *E. coli* growth were about 256 and 512 μg/mL, respectively. At a concentration of 512 μg/mL, the Zn/CFRs presented larger antimicrobial rates against *S. aureus* (74%) than *E. coli* (57%). And Zn/CFRs achieved a 77% inhibition rate against *E. coli* and completely suppressed *S. aureus* growth at a concentration of 1024 μg/mL. Zn/CFRs demonstrated effective, though not exceptional antibacterial activity [49]. The release profile of Zn^2+^ from Zn/CFRs was investigated *via* ICP-MS. In Supplementary Figure S6, the cumulative Zn^2+^ release from Zn/CFRs reached approximately 0.8% (0.03 ppm) within the initial 12 h, followed by sustained release for up to 72 h. Huang *et al.* [21] developed the Zn^2+^-based hybrid nanoparticles with Zn^2+^ release amount ranging from 10% to 20% within 4 h, which was significantly higher than ZnO nanoparticles that barely released Zn^2+^ in PBS buffer (pH 7.4). Ju *et al.* [22] prepared Zn^2+^ incorporated polysaccharide microspheres, which showed Zn^2+^ release amounts ranging from 0.5 to 1.5 ppm during the 24–72 h period. Compared with the previous literatures, the sustained low release amount of Zn^2+^ of the Zn/CFRs was attributed to the formation of catechol complexes with Zn^2+^, which hinders their rapid release and consequently diminishes the antibacterial efficacy [21, 22]. We further assessed the concentration-dependent cytotoxicity of Zn/CFRs toward L929 mouse fibroblasts via a CCK-8 assay [49, 50]. The relative cell viability of Zn/CFRs at various concentrations maintained over 99%, after culturing for 24 h (Figure 3C). This finding implied that Zn/CFRs had outstanding cytocompatibility. Figure 3D displays the DPPH scavenging ability of Zn/CFRs at various concentrations. At a concentration of 2048 μg/mL, Zn/CFRs exhibited a high DPPH scavenging efficiency of 63%.

AFM was utilized with a Zn/CFR probe to investigate the adhesion force between Zn/CFRs and base materials (mica sheet) during the testing. Figure 3E shows a schematic diagram of the AFM force measurements. The Zn/CFR-coated cantilever moved toward and away from the mica sheet at a rate of 1 μm/s, with continuous recording of the cantilever deflection throughout the process. As shown in Figure 3F, the diagram of force–displacement illustrates the adhesion force values and represents the largest force required to separate the probe from the mica sheet during retraction [51, 52]. A notable jumping-out behavior is observed as the Zn/CFR-functionalized cantilever retracts from the surface of the mica sheet. These results indicated that the Zn/CFRs exhibited good adhesion to mica sheets, suggesting their promising potential for enhancing the adhesive properties of hydrogels.

The dispersion and stability of Zn/CFRs in the hydrogel precursor solution were critical factors influencing the performance of the composite hydrogels. Figure 3G shows the digital photographs and optical microscope images of CSMA solution containing Zn/CFRs. The Zn/CFRs were uniformly dispersed in the CSMA solution to maintain a homogeneous distribution. Moreover, optical microscopy revealed that some particles around 2 μm in size were evenly distributed in the CSMA solution with no significant aggregation. Figure 3H shows the stability of the Zn/CFRs in a CSMA solution under multiple light sources. Transmittance curves were obtained after one cycle of scanning, as a function of height, with Δ*T* indicating the variation from the initial curve [53, 54]. The Δ*T* values remained close to zero over 24 h for the mixed solutions at different heights, illustrating the great stability of the Zn/CFRs in the CSMA solution [53, 54]. These findings suggested that Zn/CFRs can be uniformly distributed within hybrid hydrogels, ensuring their effectiveness in practical applications.

### Fabrication and characterization of CH-ZnCFR hydrogels

**Figure 4.**
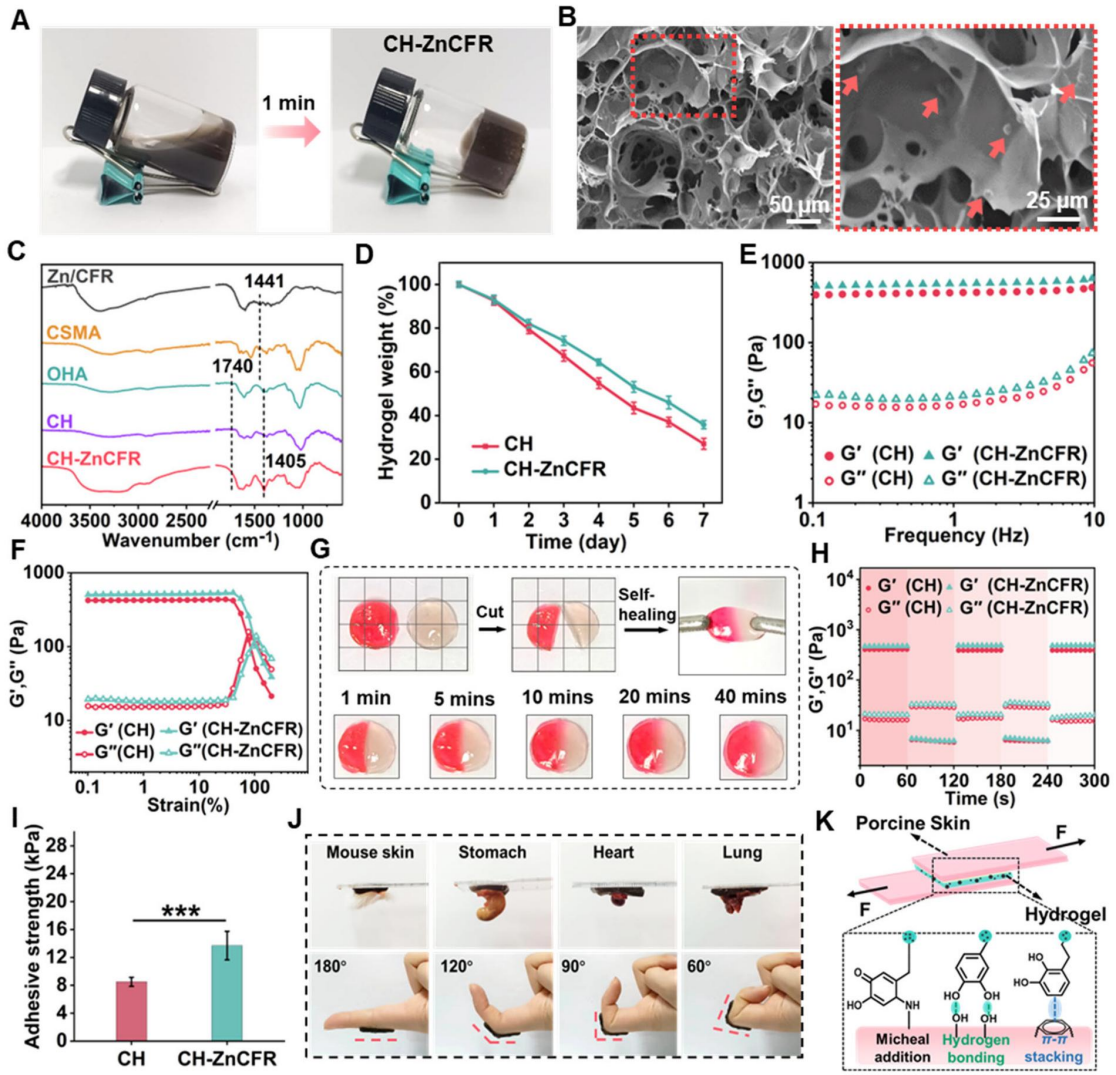
Preparation and characterization of the CH-ZnCFR hydrogel. (**A**) Preparation process of the CH-ZnCFR hydrogel. (**B**) SEM and enlarged images showing Zn/CFR (red arrows)-dispersed on the pore walls. (**C**) FTIR spectra of CH and CH-ZnCFR hydrogels. (**D**) *In vitro* degradation rate. (**E**) Rheological frequency sweeps and (**F**) strain–sweep curves of the hydrogels. (**G**) Self-healing photographs of the CH-ZnCFR hydrogel. (**H**) Dynamic step–strain curves of the hydrogels at low strain (1%) and high strain (400%). (**I**) Adhesive strength of the hydrogels to porcine skin and (**J**) photographs of CH-ZnCFR hydrogels-adhered to various biological tissues. (**K**) Possible interactions between the hydrogel and the nature skin.

To fabricate a wound dressing that manages the wound microenvironment, polysaccharide-based hydrogels (CH) were created and developed (Figure 4A), using oxidized hyaluronic acid (OHA) and methacrylamide chitosan (CSMA). ^1^H NMR and FTIR spectroscopic analyses confirmed the compositions of OHA and CSMA (Supplementary Figures S7 and S8). Considering the proper antibacterial properties and good dispersion and stability of the Zn/CFR microspheres, the polysaccharide solution containing Zn/CFRs (0.6 mg/mL) was prepared to obtain the composite hydrogels (CH-ZnCFR). The CH-ZnCFR hydrogels were formed via a dual-network strategy involving photo-crosslinking of CSMA and Schiff-base reactions between the amino groups of CMCS and the aldehyde groups of OHA. SEM images of the CH-ZnCFR hydrogels (Figure 4B) revealed a characteristic interconnected and porous microstructure, wherein Zn/CFR (red arrows)-dispersed on the pore walls. The average pore size of CH-ZnCFR hydrogels was about 40 μm (Supplementary Figure S9), indicating the negligible effect of Zn/CFRs on the porous structure of hydrogels. FTIR spectra of the CH hydrogels presented characteristic peaks of CSMA and OHA. The characteristic FTIR peak of OHA at 1730 cm^−1^ due to the C=O stretching vibration of aldehydic carbonyl groups was unobserved, a peak at 892 cm^−1^ corresponding to the hemiacetal structure was observed in CH hydrogel suggesting the formation of Schiff-base bonds between aldehyde groups of OHA and amino groups of CMCS (Figure 4C) [55]. With the addition of Zn/CFRs, the peaks at 1441 cm^−1^ corresponding to characteristic bending vibrations of benzene rings shifted to a lower position, suggesting the interaction between Zn/CFRs and the hydrogel matrix. Compared with the CH hydrogel, the CH-ZnCFR hydrogels exhibited a relative lower swelling ratio of ca. 17.7 g/g and an increased water retention capacity (Supplementary Figure S10). The degradation behavior of CH-ZnCFR is shown in Figure 4D. During the 7 days, the mass of both hydrogels decreased. With the addition of Zn/CFR microspheres, the hydrogel showed a slower degradation rate, making it suitable for use as a wound dressing.

Rheological properties and self-healing properties are crucial factors in evaluating the wound adaptability of hydrogel dressings. Figure 4E and F illustrates that all the hydrogel groups displayed elastic properties, with the storage modulus (*G*′) surpassing the loss modulus (*G*″). *G*′ of CH-ZnCFR hydrogels was greater than that of the CH hydrogels, indicating that the CH-ZnCFR hydrogels exhibited good elastic behavior. Self-healing capacity is a key factor influencing the wound suitability of hydrogel dressings [55]. To assess the self-healing property of CH-ZnCFR hydrogels, those stained with or without rhodamine-B were cut in half and then re-joined at the differently strained sections. The hydrogels were integrated after 40 min and could withstand tensile forces (Figure 4G). Moreover, the dynamic step-strain rheological tests were conducted to assess the self-healing property. As shown in Figure 4H, *G*′ of the both hydrogels decreased dramatically at high strains (400%), and rapidly recovered at low strains (1%). We propose that the Schiff-base bonds between the CSMA and OHA engage in dynamic cross-linking to enable self-healing. Meanwhile, the intermolecular hydrogen bonding between the catechol groups of Zn/CFRs and the CH matrix facilitated the faster recovery of the fractured interfaces [56].

In addition, the adhesion property was another crucial factor affecting the ability of hydrogel dressings to adapt to wounds. The adhesive strength of the CH-ZnCFR hydrogels was further determined by shear adhesion tests. As depicted in Figure 4I, the adhesive strength of the CH-ZnCFR was about 13.7 kPa, which was significantly larger than the value of the CH (8.1 kPa). The CH-ZnCFR hydrogels can adhere to various biological tissues, including the skin, stomach, heart and lung tissues (Figure 4J). Moreover, the CH-ZnCFR hydrogel can adhere to the skin of the finger joints that are bent at different angles (Figure 4J). The adhesive performance of the CH-ZnCFR was significantly enhanced, which may be ascribed to the amino groups present in nature skin tissue, which formed covalent bonds with Zn/CFRs via Michael-type addition [57]. Meanwhile, noncovalent interactions such as the π–π stacking and intermolecular hydrogen bonding also enhanced the adhesive ability of the hydrogel interfaces (Figure 4K) [35, 58].

### Effect of the Zn^2+^ release on the bioactivities of CH-ZnCFR hydrogels

**Figure 5.**
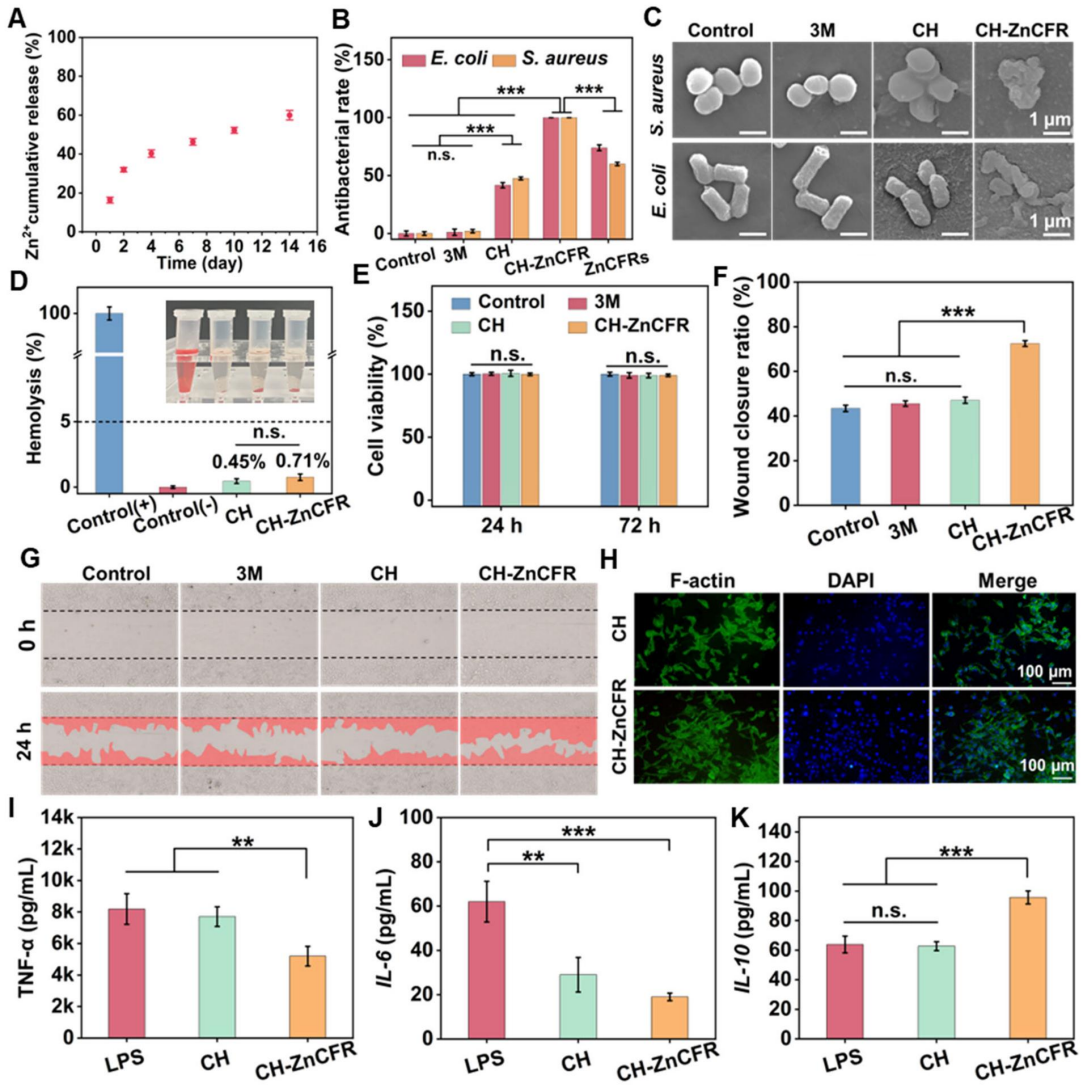
*In vitro* effects of the Zn^2+^ release on the antibacterial properties, cytocompatibility and macrophage inflammatory polarization of CH-ZnCFR hydrogels. (**A**) Zn^2+^ release profile of the CH-ZnCFR hydrogel. (**B**) Antibacterial rates. (**C**) SEM images of bacteria contacting with the hydrogels. (**D**) Hemolysis and (**E**) cell viability of fibroblasts seeded on the hydrogels. (**F**) Wound closure ratio. (**G**) Scratch assay of fibroblasts treated with different hydrogels and quantitative analysis results of scratch areas. (**H**) LCSM images of F-actin (green) and DAPI (blue) staining for cells after different treatments on day 3. (**I–K**) Corresponding *TNF-α, IL-6* and *IL-10* concentrations in cell culture supernatant of LPS-induced M1 seeded on different hydrogels were determined by ELISA.

Zn-based biomaterials with the ability of release of Zn^2+^ show good antibacterial and anti-inflammatory properties, which play important roles in tissue engineering [15, 16]. Zn^2+^ release amount from the CH-ZnCFR hydrogel was determined in PBS solution (pH = 7.2) via ICP-MS. Figure 5A demonstrates that CH-ZnCFR rapidly released Zn^2+^ ca. 20% (0.79 ppm) on the first day, followed by sustained release for up to 14 days. The released concentration on the first day was located in the minimum inhibitory concentration (MIC) range of Zn^2+^ (0.65–6.5 μg/mL) [59]. Thus, the CH-ZnCFR hydrogels is expected to inhibit *E. coli* and *S. aureus* effectively. Wu *et al.* [60] prepared Zn-doped Prussian blue frameworks, with Zn^2+^ release amounts ranging from 0.35 to 0.57 ppm within 24 h, and sustained release for 1 week. The CH-ZnCFR hydrogel exhibited a greater Zn^2+^ release within 24 h and a longer release profile than previously reported in the literature [60].

Bacterial infection is a major hindrance in wound healing [35, 60]. Hence, the antimicrobial activities of the CH-ZnCFR hydrogels against *E. coli* and *S. aureus* were determined. The antibacterial efficacy after treatment with different hydrogels is shown in Figure 5B. Compared with the commercial 3M group, the CH group presented fewer bacterial colonies and antibacterial rates of 41.6 ± 2.3% (*E. coli*) and 47.5 ± 1.3% (*S. aureus*), respectively. Notably, no colonies were observed in the CH-ZnCFR group, which presented the largest antibacterial efficiency, about 99.9% against both *E. coli* and *S. aureus*. SEM images in Figure 5C displayed the morphological changes of the bacteria contacting with the hydrogels. 3M groups showed an intact morphology, CH groups presented bacterial shrinkage and CH-ZnCFR groups showed severe rupture and leakage of contents. The antibacterial activity primarily stemmed from the synergistic effects of the Zn/CFR microparticles and CH matrix. CH-ZnCFR hydrogels enable the sustained release of Zn^2+^, which can disrupt bacterial enzyme systems and cell walls, resulting in long-term antibacterial effects [16, 39, 61]. Moreover, cations from CSMA interact with the bacterial membrane, increasing membrane permeability [57, 62]. The phenolic hydroxyl group on Zn/CFRs can interfere with bacterial metabolism by chelating metal ions on the cell membrane, thereby inhibiting bacterial growth [35]. Moreover, CH-ZnCFR exhibited a DPPH clearance ability (22.3%), which was ca. 10 times greater than that of CH hydrogel (Supplementary Figure S11). The phenolic hydroxyl groups of CFR are strong free radical terminators, endowing hydrogels with certain antioxidant abilities [34, 35].

Good biocompatibility is also essential for hydrogels to effectively promote wound repair [60, 61]. Hemolysis and cytocompatibility of CH-ZnCFR hydrogels were evaluated *in vitro*. As depicted in Figure 5D, both the CH and CH-ZnCFR hydrogels exhibited hemolysis rates below 5%. The CH-ZnCFR hydrogel exhibited no evident hemolysis. Besides, the CH-ZnCFR hydrogel maintained over 90% cell viability after being incubated with L929 cells for 24 and 72 h (Figure 5E). We conducted a scratch assay to further investigate the impact of various samples on cell migration. As shown in Figure 5F and G, after incubation with the CH-ZnCFR hydrogel extract for 24 h, the cell migration rate reached 73.5 ± 1.3%, which significantly surpassed that of the other groups. The CH-ZnCFR hydrogel increased cell migration, which is correspondence with the previous research [61, 63]. Researchers have shown that high concentrations (>5.2 ppm) of released Zn^2+^ can induce significant cytotoxic effects, whereas minimal quantities of Zn^2+^ can increase cell migration [61, 63]. Notably, the cumulative amount of Zn^2+^ released was ca. 60% (2.9 ppm) at 14 days (Figure 5A), which remained below the cytotoxic threshold (>5.2 ppm) reported in the literature [39]. The morphology of cells incubated with the membranes was further observed by the fluorescence microscopy. As shown in Figure 5H, the fibroblasts cultured on the hydrogels displayed an expanded cell morphology. Overall, the developed hydrogel exhibited excellent biocompatibility.

Zn^2+^ have the ability of transforming proinflammatory M1 phenotype to M2 phenotype. The maintenance and transformation of macrophages phenotypes of the CH-ZnCFR hydrogels were confirmed by measuring the levels of inflammatory cytokines (*TNF-α* and *IL-6*) and anti-inflammatory cytokines (*IL-10*) in the cell culture supernatant using ELISA [64]. As shown in Figure 5I, the level of *TNF-α* in the CH-ZnCFR groups, decreased to about 5.2 ng/mL, compared to the control (8.1 ng/mL) and the CH groups (7.7 ng/mL) (*P* < 0.01). Meanwhile, the level of *IL-6* in the hydrogel groups, decreased almost 1-fold (CH 29 pg/mL, CH-ZnCFR 19 pg/mL) compared to the control group (62 pg/mL) (Figure 5J). On the contrary, the level of *IL-10* in the hydrogel groups, especially the CH-ZnCFR groups (95.6 pg/mL) increased around 50% than that of the control group (63.8 pg/mL) and the CH group (62.7 pg/mL) (*P* < 0.001) (Figure 5K). The results showed that CH-ZnCFR hydrogels due to the addition of Zn/CFR could down-regulate the inflammatory cytokines (*TNF-α* and *IL-6*), up-regulate anti-inflammatory cytokines *(IL-10*) and promote the transformation of M1 into M2, thereby enhancing the immunocompromise by modulating M2.

### Wound healing and tissue immunological analysis of infected wounds

**Figure 6.**
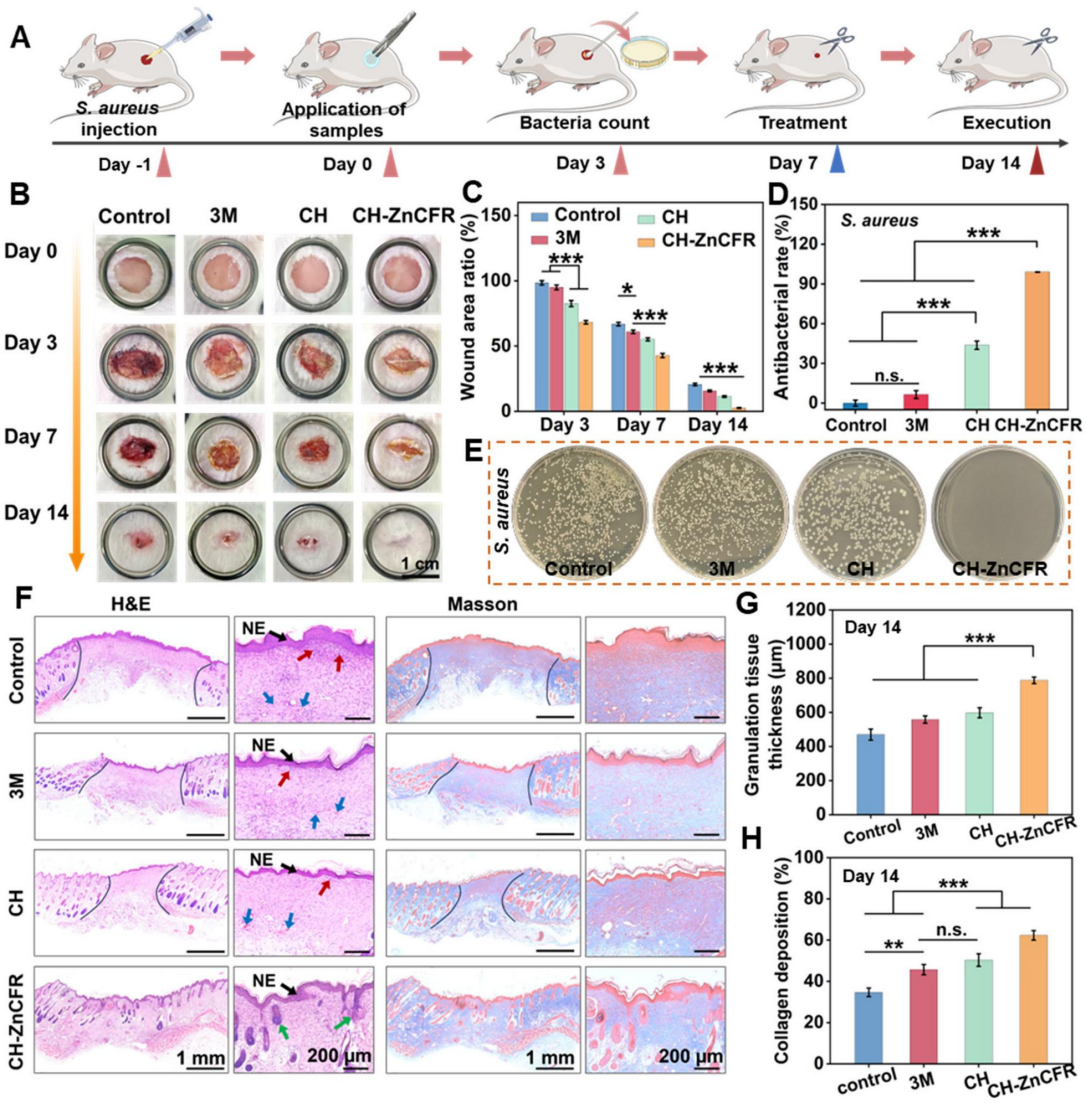
*In vivo* assessment of wound healing in *Staphylococcus aureus*-infected wounds. (**A**) Schematic illustration of the establishment of the *S. aureus*-infected wound model and treatment timeline. (**B**) Representative photos of wounds after implantation of different hydrogels. (**C**) Wound area ratio in different groups. (**D**) The antibacterial rates of different hydrogels in the wound tissue. (**E**) Representative images of *S. aureus* clones on LB plates after different treatments. (**F**) H&E and M&T staining images of regenerated skin tissue on day 14 (NE, new epithelization; red arrows, inflammatory cells; blue arrow, red blood cells; green blood, hair follicles). (**G**) Quantitative analysis of granulation tissue thickness on day 14. (**H**) Quantitative analysis of collagen deposition on day 14.

Considering the excellent antimicrobial properties and cytocompatibility of the CH-ZnCFR hydrogels *in vitro*, we further investigated their therapeutic efficacy *in vivo*. A full-thickness *S. aureus*-infected wound model was established to assess the healing potential of the CH-ZnCFR hydrogels (Figure 6A). The wounds were treated with sterile gauze (control), 3M Tegaderm™ dressing, CH hydrogel or CH-ZnCFR hydrogel. After 3 days of treatment, pus remained at the wound site in the control group (Figure 6B) and was quantified through standard swab culture and agar plate counts, verifying the successful establishment of bacterial infection within the entire skin defect. The CH-ZnCFR hydrogel exhibited greater antibacterial efficacy (99%) than the CH hydrogel (43%) *in vivo* (Figure 6D and E). Moreover, at the same healing time point, wounds treated with the CH-ZnCFR hydrogel were significantly smaller than those treated with the 3M Tegaderm™ dressing and the other groups (Figure 6B). Strikingly, wounds in the CH-ZnCFR group showed almost entirely healing after 14 days of treatment, with less than 3% of the wound area remaining (Figure 6C). This phenomenon can be attributed to the early-stage antimicrobial effects facilitated by the Zn/CFRs and chitosan matrix, along with the promotion of fibroblast migration induced by Zn^2+^ during the wound healing process.

Simultaneously, histological evaluations of the wound healing process were conducted through H&E and M&T staining. According to the H&E-stained images, the granulation tissue thicknesses of the control, 3M, CH and CH-ZnCFR hydrogel groups were measured as 470, 558, 598 and 788 μm, respectively (Figure 6F and G). Compared with the other three groups, the CH-ZnCFR group exhibited thicker new epithelization (black arrow), fewer inflammatory cells (red arrow), fewer red blood cells (blue arrow) and more mature skin appendages, such as hair follicles (green arrow). Further assessment of collagen deposition and arrangement through M&T staining revealed the accumulation of muscle fibers in the control and 3M groups, with irregularly arranged collagen fibers (Figure 6F and H). Conversely, the CH and CH-ZnCFR groups presented greater collagen deposition, with the CH-ZnCFR group showing denser and more orderly collagen fibers, indicating improved ECM and tissue remodeling. These results suggested that the CH-ZnCFR was most effective at promoting wound healing due to the capacity of the Zn^2+^ to not only eliminate bacteria effectively but also promote fibroblast migration, thereby efficiently stimulating processes such as re-epithelialization and collagen deposition.

**Figure 7.**
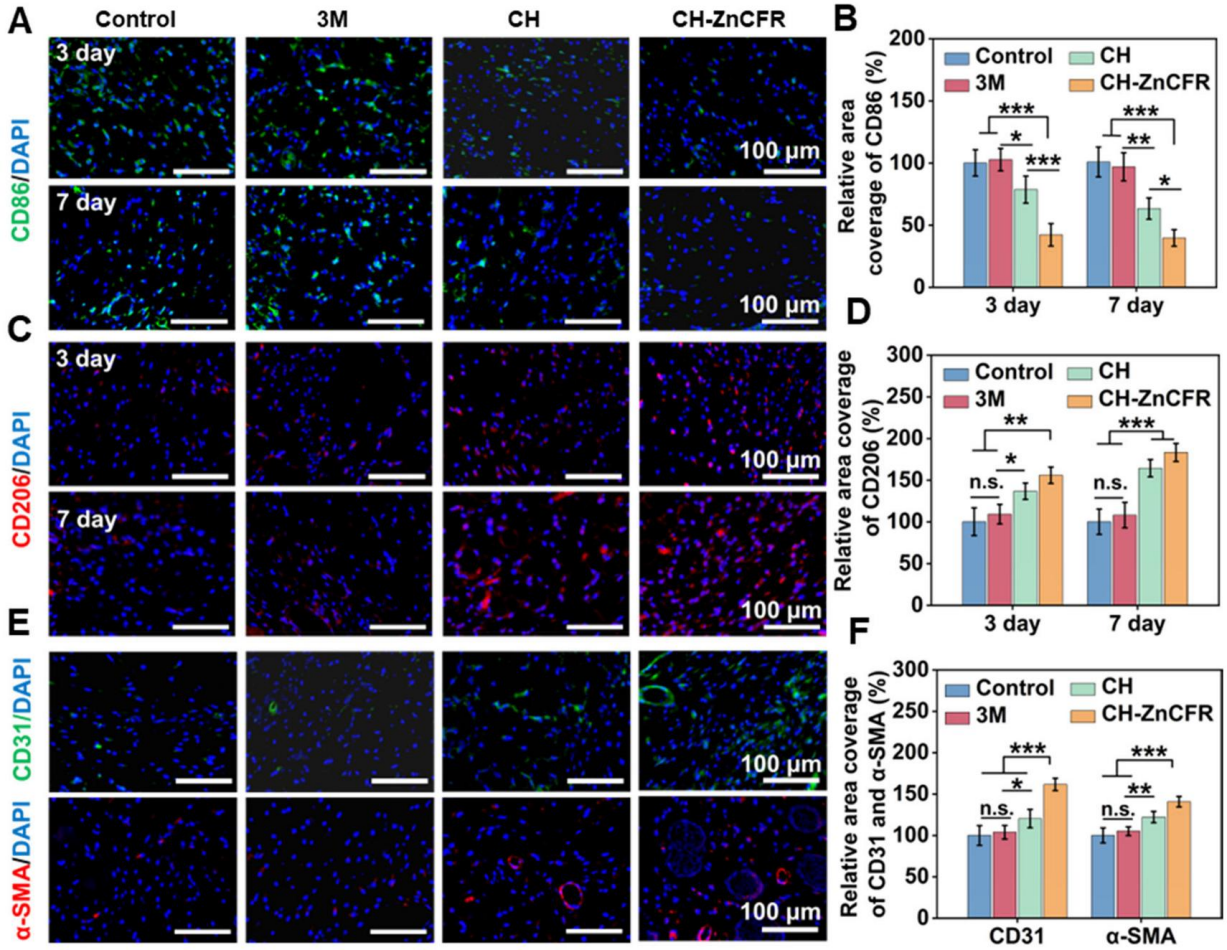
*In vivo* anti-inflammatory and neovascularization assessment of the hydrogels. Immunofluorescence staining images of (**A**) CD86, (**C**) CD206 and (**E**) *α*-SMA, CD31. Quantitative results of (**B**) CD86, (**D**) CD206 and (**F**) *α*-SMA, CD31expression.

Bacterial infections that induce severe inflammatory responses can hinder the wound healing process [56]. Zn^2+^ have shown effects on macrophage polarization, thereby reducing inflammation and facilitating wound repair [63, 64]. Thus, immunofluorescence staining was performed to assess the M1/M2 balance in CH-ZnCFR hydrogel-treated wounds, with CD86 (green) marking M1 macrophages and CD206 (red) marking M2 macrophages [57]. At days 3 and 7, immunofluorescence staining (Figure 7A and C) revealed that CD86 fluorescence was highest on day 3 and gradually decreased over time, whereas CD206 expression displayed the opposite trend. Quantitative analysis (Figure 7B and D) revealed that in the CH-ZnCFR group, the CD86 expression level was notably lower compared to the other three groups, while the CD206 expression level was notably higher compared to the other groups. Concurrently, immunofluorescence staining of the angiogenesis markers CD31 (green) and *α*-SMA (red) was employed to assess the neovascularization formation capacity of the CH-ZnCFR hydrogels [56]. As shown in Figure 7E and F, the immunofluorescence images and quantitative analysis of CD31 and *α*-SMA on day 14 revealed that their fluorescence expression levels were follows: CH-ZnCFR > CH > 3M > Control. The levels of circular structures with red or green fluorescence in the CH-ZnCFR group were significantly greater than those in the other groups, indicating an earlier and stronger activation of the angiogenic responses [34].

Moreover, compared with the control group, the coagulation time in the CH-ZnCFR group was reduced by 98 s and the amount of blood loss was decreased by about 50% in a mouse tail amputation model (Supplementary Figure S12). The hemostatic effectiveness of CH-ZnCFR is attributed to two primary mechanisms: (1) electrostatic interactions between chitosan and erythrocytes or platelets [65], and (2) the covalent bonding of catechol groups with blood proteins, such as fibrinogen and fibrin, which collectively facilitate the rapid formation of a physical barrier [65, 66]. Taken together, these results suggested that the CH-ZnCFR sample had an exceptional capacity to inhibit bleeding, bacterial reproduction and induce macrophage polarization and angiogenesis, thus promoting the healing of bacterially infected wounds.

## Conclusion

In summary, heterogeneous Zn/CFRs with a Zn^2+^-enriched surface and internal ZnO nanoparticles were successfully prepared *via* a one-pot hydrothermal method. By optimizing synthesis conditions, the controllable synthesis method of Zn/CFRs was achieved and the formation mechanism of Zn/CFRs was elucidated. Notably, owing to the unique microstructure of the as-prepared Zn/CFRs, CH-ZnCFR composite hydrogels were able to rapidly release Zn^2+^ in the initial phase and sustain the release of Zn^2+^ for 14 days. CH-ZnCFR effectively inhibited *S. aureus* proliferation, scavenged DPPH and facilitated the macrophage polarization *in vitro*. Moreover, in a chronic infected wound healing model, CH-ZnCFR composite hydrogels exhibited the regenerative potential by reducing inflammation, enhancing collagen deposition, increasing granulation regeneration and angiogenesis. The CH-ZnCFR composite hydrogels represent a promising strategy for treating chronic wounds.

## Supplementary Material

rbaf081_Supplementary_Data

## Data Availability

All the data are available from the corresponding authors upon reasonable request.
