# Supplementary material for: Heterogeneous zinc/catechol-derived resin microsphere-functionalized composite hydrogels with antibacterial and anti-inflammatory activities promote bacterial-infected wound healing
Source: Regen Biomater. 2025 Aug 9;12:rbaf081. doi: 10.1093/rb/rbaf081 (PMC12417082; doi:10.1093/rb/rbaf081)
Supplement: rbaf081_Supplementary_Data [file rbaf081_supplementary_data.docx]

SUPPORTING INFORMATION

for

**Heterogeneous zinc/catechol-derived resin microsphere-functionalized composite hydrogels with antibacterial and anti-inflammatory activities promote bacterial-infected wound healing**

Lianyi Qu^1†,^, Anle Yang^1†^, Yulei Shi^1^, Jianglong Liu^1^, Xueyan Li^1^, Bohan Mao^1^, Xiaoran Li^2^, Fang Zhou^1*^, Yingjun Xu^1*^

^1^ Institute of Functional Textiles and Advanced Materials, College of Textiles & Clothing, Qingdao Key Laboratory of Flame-Retardant Textile Materials, State Key Laboratory of Bio-fibers and Eco-textiles, Qingdao University, Qingdao 266071, China.

^2^ Innovation Center for Textile Science and Technology, College of Textiles, Donghua University, Shanghai 201620, China

Lian-Yi Qu and An-Le Yang contributed equally to this work.

Corresponding to fangzhou@qdu.edu.cn (FZ); yingjun.xu@qdu.edu.cn (YJX)

**1. Method**

*1.1. Synthesis and characterization of OHA and CSMA*

The synthesis of the aldehyde hyaluronic acid (OHA) was conducted according to the references [1]. Briefly, 1 g of HA was dissolved in 100 mL of deionized water and combined with 5 mL of sodium periodate solution under dark conditions for 2 hours. Subsequently, 0.5 mL of ethylene glycol was introduced to neutralize any remaining unreacted sodium periodate. Following the reaction, the resulting solution underwent a 5-day dialysis process and subsequently freeze-dried to yield purified OHA.

CSMA was synthesized by a one-step N-acylation reaction between the amino group of chitosan and methacrylic anhydride [2]. In brief, 1 g of chitosan was dissolved in acetic acid (2%) and mixed thoroughly before the addition of 3 mL of methacrylic anhydride (94%). The resulting mixture was allowed to react for 6 hours at a temperature of 60°C. Subsequently, the CSMA solution was neutralized using NaHCO_3_ (10 w/v%) until reaching a pH of 6.5. To purify the product, the solution was dialyzed for a period of 5 days and followed by freeze-drying. The chemical structures of the OHA and ChMA were assessed by ^1^H nuclear magnetic resonance (^1^H NMR, Bruker, Germany) and Fourier transform infrared spectrometer (FTIR, Thermo Scientific, USA).

*1.2. Characterization of Zn/CFRs*

The morphological structure of the Zn/CFR samples was determined by scanning electronic microscope (SEM) (TESCAN, Czech), field-emission SEM (FESEM) (TESCAN MIRA LMS, Czech) and transmission electron microscopy (TEM) (Gatan, USA). The hydrodynamic diameter and zeta potential of the Zn/CFR samples were determined with a Nano ZSE (Malvern, UK) at 25 °C. The elemental compositions of the Zn/CFRs were determined via energy-dispersive X-ray spectroscopy (EDX) (Oxford Instruments, UK) coupled with the FESEM. X-ray powder diffraction (XRD) was tested with an Ultima IV instrument (Rigaku, Japan) using Cu Kα radiation. X-ray photoelectron spectroscopy (XPS) was performed with Al Kα excitation radiation on an ESCALAB 250Xi apparatus (Thermo Fisher Scientific, USA). The chemical structure of Zn/CFRs was assessed by Fourier transform infrared spectroscopy (FTIR, Thermo Scientific, USA) and solid-state ^13^C nuclear magnetic resonance spectroscopy (^13^C NMR, Bruker, Germany), respectively. The adhesion force of Zn/CFRs was measured by atomic force microscope (AFM) (Bruker, Germany) in the tapping mode according to some recent studies [3]. The L929 mouse cell viability assay test for the Zn/CFRs was rated by the CCK-8 assay followed by a procedure according to previous works [4]. The dispersion of Zn/CFRs in chitosan solutions was observed under an SG optical microscope (Suzhou Shenying Optical Co., Ltd., China). The dispersive stability of Zn/CFRs microspheres in chitosan solutions was tested on a TOWER Multiple light scatterers (Formulacation, France) by scanning the bottle using an 880 nm near-infrared light at a horizontal height of 3-35 mm every half hour for 24 h.

*1.4 Release behavior of Zn^2+^*

Zn^2+^ release amounts from the Zn/CFRs were tested by inductively coupled plasma atomic emission spectrometry (ICP-MS, Agilent 7800, USA). Briefly, 5 mL of Zn/CFR (512 μg/mL) PBS solution (pH 7.4) was placed in a dialysis bag and dialyzed against 25 mL of deionized water. The dialysate was collected every 4 h during the first 12 h, and every 12 h from 12 to 72 h. After each collection, the external solution was replaced with 25 mL of fresh deionized water. The collected dialysate samples were then analyzed for Zn content using an ICP-MS.

Zn^2+^ release amounts from the CH-ZnCFR hydrogels were also tested by ICP-MS. Briefly, freeze-dried hydrogel samples were prepared by cutting them into circular pieces with a diameter of 1 cm. These hydrogel pieces were then placed into dialysis bags and immersed in 30 mL PBS with a pH of 7.4. At predetermined time intervals, 6 mL of the release medium was collected and replaced with an equal volume of fresh PBS. The collected solutions were subjected to analysis using ICP-MS to determine the concentration of Zn^2+^.

*1.5 Swelling and water retention capabilities*

The swelling and water retention capabilities of the developed CH-ZnCFR hydrogels were investigated by the weighing method [5]. The freeze-dried hydrogel samples were cut into discs with a diameter 10 mm, precisely weighted (W_d_) and then immersed in phosphate-buffered saline (PBS) at 37°C. At determined intervals, hydrogel specimens were withdrawn and weighed (W_w_). When the hydrogel weight no longer varies, the operation was stopped. The swelling ratio was calculated as follows.

Swelling ratio (%) = (W_w_ - W_d_)/W_d_ 🞨100% (1)

For water retention capacity assay, the CH-ZnCFR hydrogels in the swelling equilibrium state (W_0_) were transferred to a 37 °C oven. The weight of hydrogels at various time intervals were measured and record (W_t_). The water retention was determined by the following equation.

Water retention (%) = (W_t_/W_0_) × 100% (2)

*1.6 The degradation behavior of hydrogels*

The freeze-dried hydrogels (W_s_), were submerged in PBS at 37 °C. After a predetermined interval, the hydrogels were extracted, freeze-dried, and weighed (W_t_). The degradation ratio (%) was calculated as the formula.

Degradation ratio (%) = W_t_/W_s_ × 100% (3)

*1.7 Rheological properties*

The Rheological properties of the CH-ZnCFR hydrogels were conducted at 37 °C utilizing an MCR302 rheometer (Anton Paar, Austria). The test modes were as follows: (a) strain sweep assay was performed with oscillatory strain increasing from 0.1% to 400%; (b) frequency sweep test was performed with the frequency increasing from 0.1 Hz to 10 Hz; (c) dynamic step-strain sweep tests was used to assess the self-healing capabilities, with oscillatory strains alternating from small strains (1%) to large strains (400%).

*1.8 Adhesion strength of hydrogels*

The adhesion strength of hydrogels was evaluated by a lap-shear test using a universal materials testing machine (Instron 5300, USA) equipped with a 100 N load cell [6]. Each hydrogel specimen (10 mm × 10 mm × 1 mm) was positioned between two fresh pigskin pieces, with a test speed set at 10 mm/min. Adhesive strength was determined by dividing the maximum load recorded by the adhesive area.

*1.9 Antibacterial properties of Zn/CFR microspheres and CH-ZnCFR hydrogels*

The antibacterial properties of the Zn/CFRs against *E. coli* (CMCC(B)44102) and *S. aureus* (CMCC(B)26003) was determined by the broth microdilution method. Standard strains of *E. coli* and *S. aureus* were provided by Beijing Microbiological Culture Collection Center (Beijing, China). Briefly, the bacterial suspension (10^6^ CFU/mL) was mixed with various concentrations of Zn/CFRs samples and then incubated in a sharking incubator for 24 h at 37 °C. As a control, MH broth was used instead of Zn/CFRs solution. The lowest concentration of Zn/CFRs in the suspension that can inhibit the growth of a microorganism (the minimum inhibitory concentration, MIC) after 12 h incubation was tested and analyzed. 100 μL of the suspension with various concentrations of Zn/CFRs was taken and dispersed evenly on agar plates, and incubated for 12 h. The antimicrobial rates of Zn/CFRs were calculated.

Given the disparities in size and morphology between nanoparticles and hydrogels, the antibacterial properties of different hydrogels against *E. coli* and *S. aureus* were assessed by the colony count method. In brief, the freeze-dried hydrogel was cut into 1 cm diameter discs, sterilized under UV light, and placed in 5 mL sterile centrifuge tubes. 25 μL of bacterial suspension (10^8^ CFU/mL) and 2 mL of sterilized PBS solution were subsequently added and inoculated with each sample. The mixture was then incubated in shaking flasks (37 ^o^C, 250 rpm, 24 h). Then, 100 μL of the bacterial suspension was taken in drops on agar plates, dispersed evenly with a coating rod, and grown at 37 ^o^C for 12 h. All colony units were photographed and counted for quantitative analysis of the antimicrobial efficacy of hydrogels. Subsequently, the bacteria after different treatments were dehydrated and observed by SEM.

*1.10 Antioxidant properties of Zn/CFR microspheres and CH-ZnCFR hydrogels*

The antioxidant abilities of *Zn/CFR* microspheres and *CH-ZnCFR hydrogels* were evaluated by the DPPH assay. Briefly, 3 mL of Zn/CFR/DPPH mixed solutions different Zn/CFR concentrations were configured. Equal volume of 0.1 mM DPPH solution was used as control. The above mixture was incubated in the dark for 1 h. Moreover, freeze-dried hydrogel samples (10 mg) were incubated with 3 mL of 95% ethanol solution containing 0.1 mM DPPH in a dark environment for 1 h. Subsequently, 100 µL of the supernatants were extracted to measure absorbance at 517 nm.

*1.11 Hemocompatibility of the hydrogels*

The red blood cells were diluted to a concentration of 5% (*v/v*) and set aside. A volume of 500 μL of the 5% (*v/v*) red blood cell suspension was combined with 500 μL of PBS in a 1.5 mL centrifuge tube. Dry hydrogel was subsequently added to the centrifuge tube, ensuring complete immersion in the red blood cell suspension. Deionized water served as the positive control, while PBS served as the negative control. The resulting solutions were incubated at 37°C for 1 hour, followed by centrifugation at 5000 rpm for 6 minutes. Subsequently, 100 μL of the supernatant was transferred to a 96-well microplate, and the absorbance of the solution was measured at 540 nm using a spectrophotometer. The hemolysis percentage was calculated as follows:

*Hemolysis (%)* = [(*A_p_–A_b_)/(A_t_–A_b_*)] ×100% (4)

where, *A_p_* denotes the absorbance of the supernatant obtained from the hydrogel, *A_t_* represents the absorbance of the supernatant from the positive control, and *A_b_* refers to the absorbance of the supernatant from the negative control.

*1.12 In vivo hemostatic performance*

The hemostatic efficacy of the hydrogel was assessed using a mouse tail amputation model. All animal procedures adhered to the guidelines outlined in the National Research Council Guidelines for the Care and Use of Laboratory Animals and were approved by the Ethics Committee of Qingdao University School of Medicine. Mice were anesthetized with an [intraperitoneal injection](https://www.sciencedirect.com/topics/pharmacology-toxicology-and-pharmaceutical-science/intraperitoneal-injection) of 7 wt% [chloral hydrate](https://www.sciencedirect.com/topics/pharmacology-toxicology-and-pharmaceutical-science/chloral-hydrate). Subsequently, two-thirds of the tail was excised using surgical scissors, and the tail was elevated for 15s to facilitate normal hemostatic blood loss. A pre-weighed hydrogel sample (1.0 cm × 1.0 cm) was then applied to the bleeding site. After clotting was completed, the filter paper and CH-ZnCFR absorbed blood were weighed again to calculate blood loss.

**2. Results and discussion**

*2.1 characterization of Zn/CFRs*


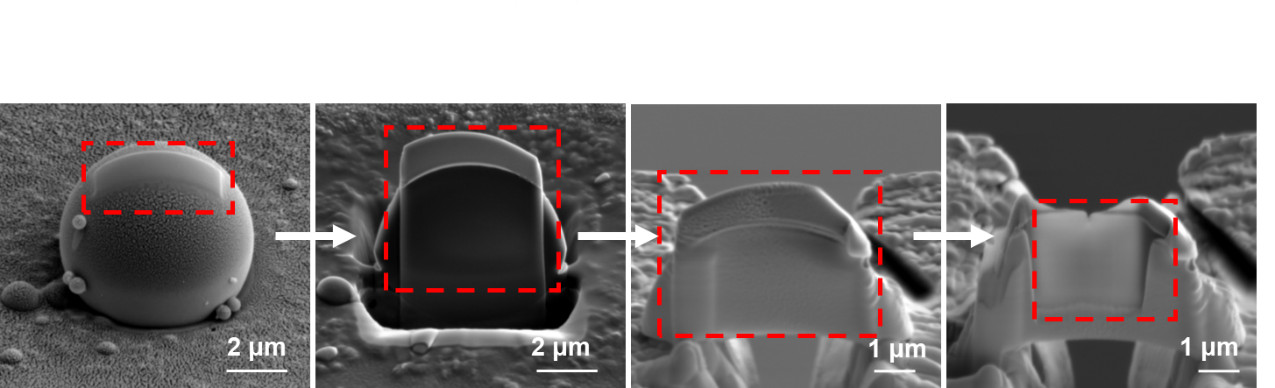


**Figure S1.** The preparation of inner surface of the Zn/CFRs using Focused Ion Beam


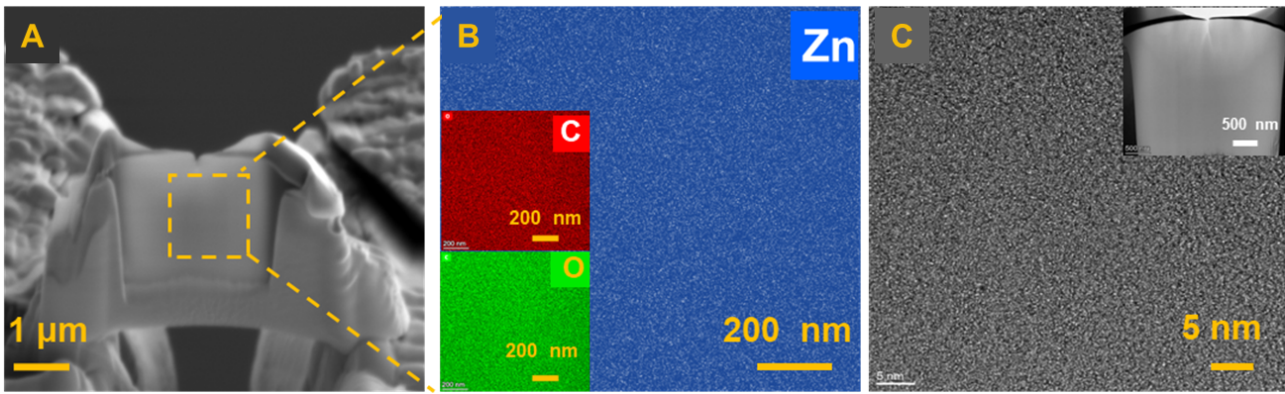


**Figure S2.** SEM (A), EDS (B) and HRTEM (C) images of inner surface of the Zn/CFRs

The chemical structure of Zn/CFRs was determined by FTIR and ^13^C NMR as depicted in Figure. S5 a and b. It can be found that the strong and broad absorption peak at 3385 cm^-1^ corresponds to the stretching vibration of -OH, while the absorption peak at 2922 cm^-1^ is attributed to the stretching vibration of C-H. The absorption peaks at 1594 cm^-1^ and 1441 cm^-1^ represent characteristic absorption bands of benzene rings. The intense absorption band at 1334 cm^-1^ is attributed to the stretching vibration of the C-O bond, and the peak at 1015 cm^-1^ belongs to the stretching vibration of -CH_2_OH. Out-of-plane bending vibrations of benzene rings occur in the range of 903-612 cm^-1^, indicating the presence of multi-substituted products of benzene rings. The NMR spectrum displays a broad peak at 28.5 ppm corresponding to the carbon atoms in the methylene group, accompanied by a shoulder peak at 16.6 ppm. The presence of peaks at 181.9 and 143.2 ppm in the ^13^C NMR spectrum can be attributed to carbon atoms belonging to the quinone and phenolic groups, respectively. Additionally, the resonance signal at 70.8 ppm confirms the existence of residual methylol groups. Above results confirm the successful synthesis of the CFR as we anticipated [7].


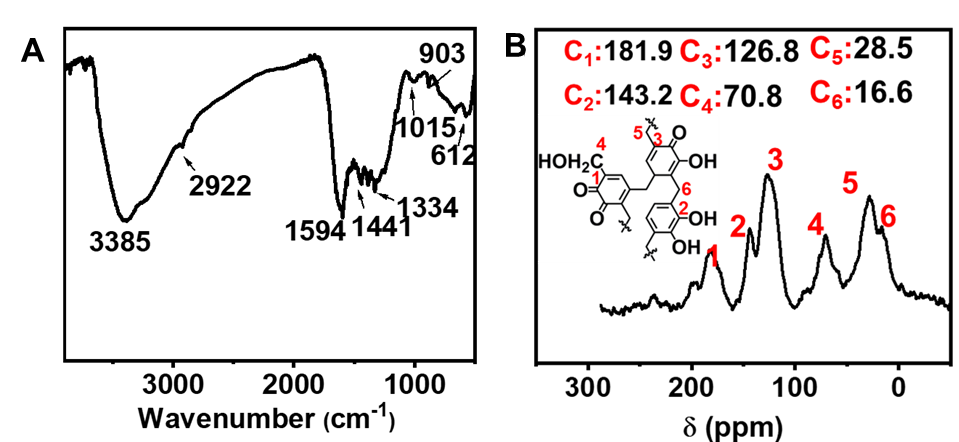


**Figure S3.** FTIR (A) and solid-state^13^ C NMR (B) spectrum spectra of the Zn/CFRs

**Figure S4.** XRD patterns of the Zn/CFRs


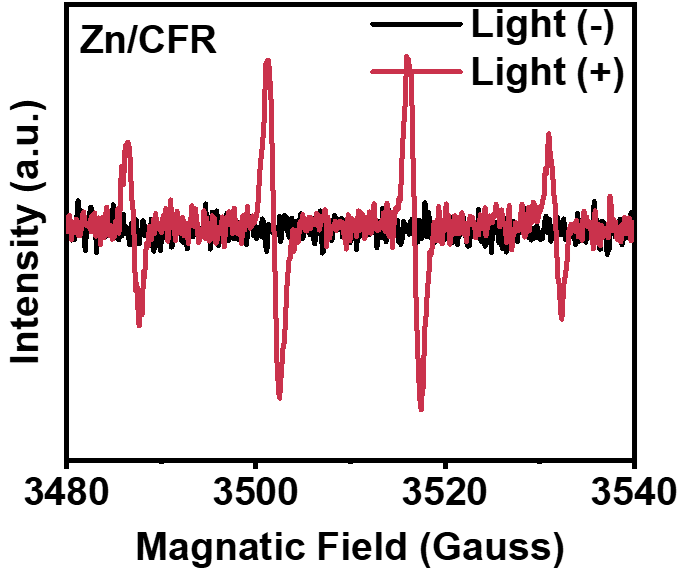


**Figure S5.** EPR spectra of the Zn/CFRs under ultraviolet irradiation

**
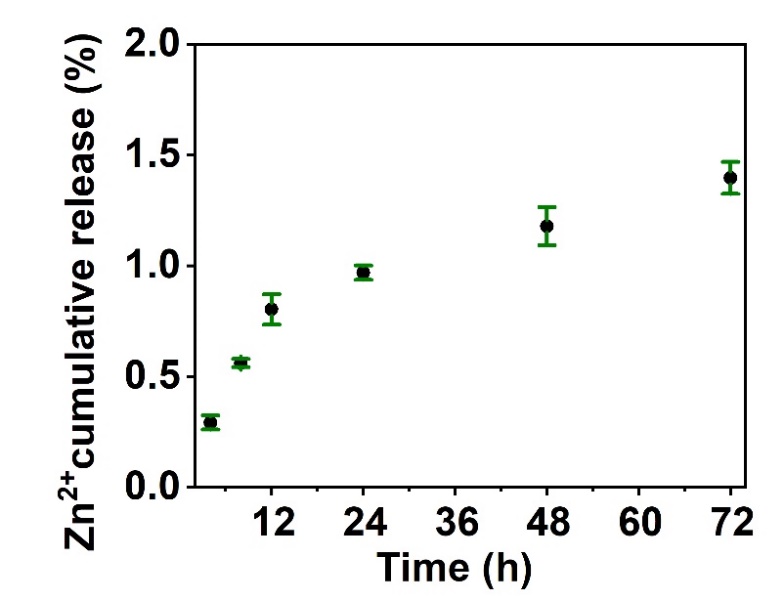
**

**Figure S6.** Zn^2+^ release profile of the Zn/CFRs

*2.2 Characterization of the CH-ZnCFR hydrogels*

As shown in Figure. S7A, ^1^H-NMR spectrum of the CSMA shows peaks at 5.28 ppm and 5.68 ppm, which are originated from the vinyl protons [2]. Meanwhile, the proton signals detected between 3.26~3.73 ppm signify the sugar backbone of chitosan. The degree of substitution of CSMA was calculated by the equation in Figure. S7a, which was about 21.7%. In FTIR spectrum of the CSMA, the prominent absorption bands at 1653 cm^−1^, 1540 cm^−1^ and 1321 cm^−1^, corresponding to the amide I, amide II and amide III bands (Figure S7B), suggested the formation of grafted methacrylate groups [8]. For the synthesis of OHA, new peaks at 1730 and 895 cm^−1^ due to the C=O stretching vibration of aldehydic carbonyl groups and hemiacetal bonds are appeared in the spectrum (Figure S8) [8]. The above results indicate the successful synthesis of CSMA and OHA.


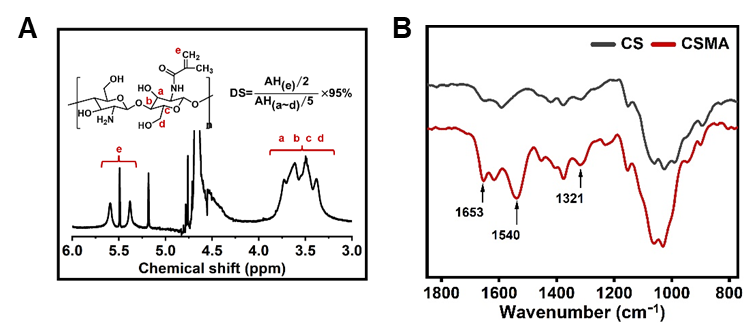


**Figure S7.** Chemical structure of the prepared CSMA. (A) ^1^H NMR spectrum of CSMA. (B) FTIR spectra of CS and CSMA.


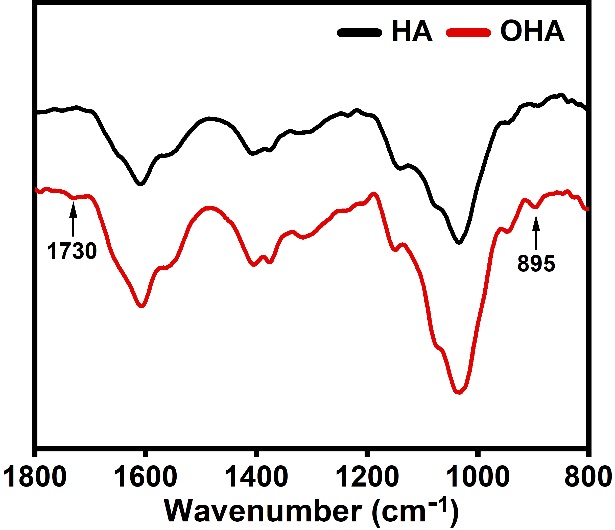


**Figure S8.** FTIR spectra of HA and OHA


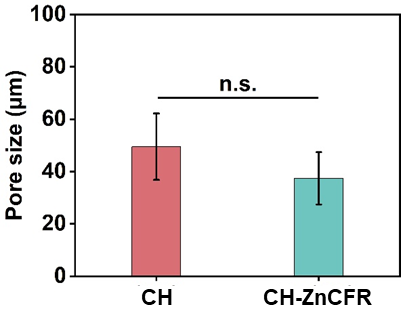


**Figure S9.** Average pore size of the CH and CH-ZnCFR hydrogels


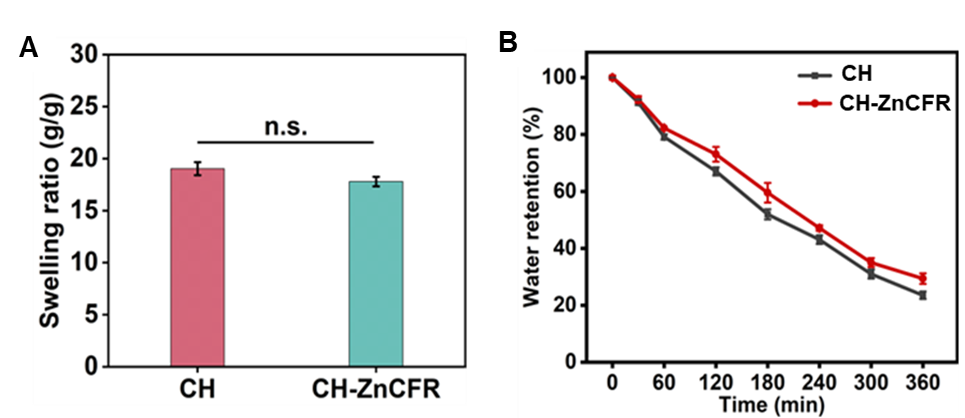


**Figure S10.** Swelling rate (A) and moisture retention (B) of the CH and CH-ZnCFR hydrogels

*
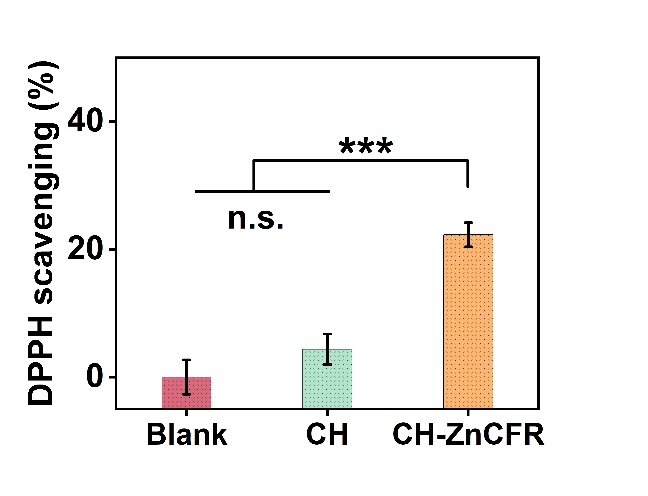
*

**Figure S11.** DPPH scavenging ratio of the CH and CH-ZnCFR hydrogels


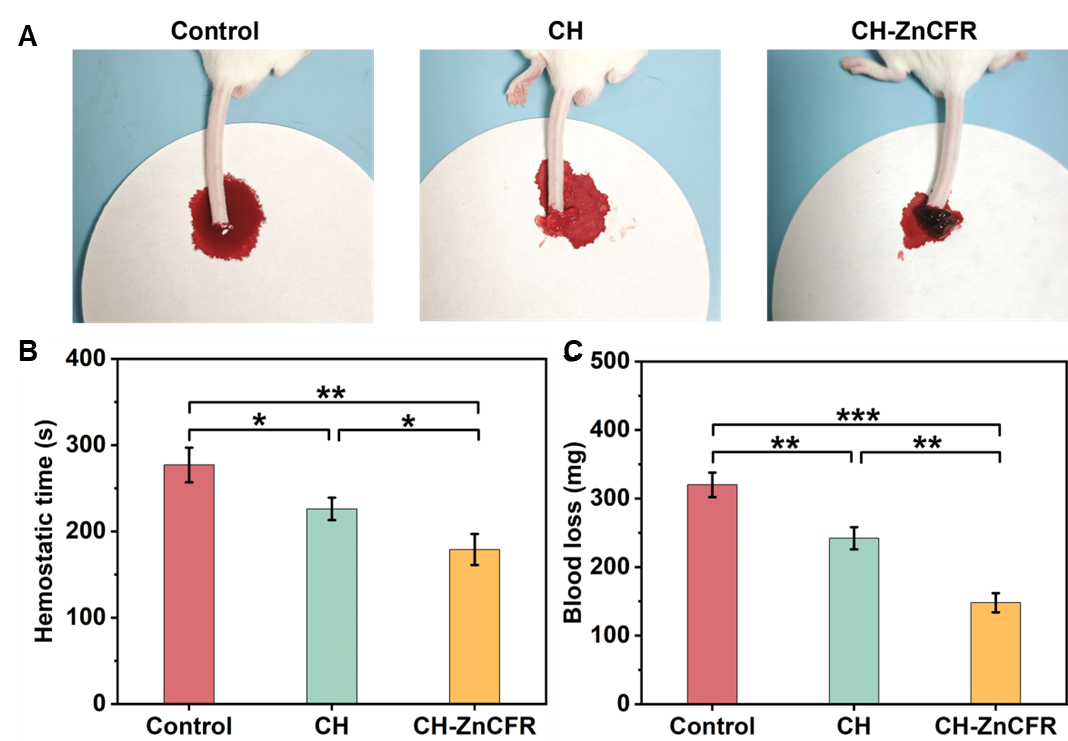


**Figure S12.** In vivo hemostatic performance of the hydrogels. (A) Hemostatic digital photographs, (B) coagulation time, and (C) blood loss in tail-amputated mice treated with different samples.

As shown in Figure S12, the CH-ZnCFR group exhibited a significant reduction in coagulation time by 98 s, compared to the Control group. Notably, both the coagulation time and blood loss in the CH-ZnCFR were significantly reduced relative to the Control and CH groups. And the blood loss in the CH-ZnCFR group (148 mg) was markedly lower than that of the CH group (242 mg).

**References**

1. Li L, Wang N, Jin X, Deng R, Nie S, Sun L, Wu Q, Wei Y, Gong C. Biodegradable and injectable in situ cross-linking chitosan-hyaluronic acid based hydrogels for postoperative adhesion prevention. *Biomaterials* 2014;35:3903-3917.

2. Li X, Sun S, Yang A, Li X, Jiang Z, Wu S, Zhou F. Dual-crosslinked methacrylamide chitosan/poly (ε-caprolactone) nanofibers sequential releasing of tannic acid and curcumin drugs for accelerating wound healing. *International Journal of Biological Macromolecules* 2023;253:127601.

3. Mao X, Gong L, Xie L, Qian H, Wang X, Zeng H. Novel Fe_3_O_4_ based superhydrophilic core-shell microspheres for breaking asphaltenes-stabilized water-in-oil emulsion. *Chemical Engineering Journal* 2019;358:869-877.

4. Song Z, Yao W, Zhang X, Dong Y, Zhang Z, Huang Y, Jing W, Sun L, Han Y, Hu F. Controlled growth of metal-organic frameworks on small intestinal submucosa for wound repair through combined antibacterial and angiogenic effects. *Nano Today* 2024;54:102060.

5. Qi X, Huang Y, You S, Xiang Y, Cai E, Mao R, Pan W, Tong X, Dong W, Ye F. Engineering robust Ag‐decorated polydopamine nano‐photothermal platforms to combat bacterial infection and prompt wound healing. *Advanced Science* 2022;9:2106015.

6. Zhao H, Hao S, Fu Q, Zhang X, Meng L, Xu F, Yang J. Ultrafast fabrication of lignin-encapsulated silica nanoparticles reinforced conductive hydrogels with high elasticity and self-adhesion for strain sensors. *Chemistry of Materials* 2022;34:5258-5272.

7. Yang Y, Ji H, Duan H, Fu Y, Xia S, Lü C. Controllable synthesis of mussel-inspired catechol-formaldehyde resin microspheres and their silver-based nanohybrids for catalytic and antibacterial applications. *Polymer Chemistry* 2019;10:4537-4550.

8. An P, Wei H, Zhang Y, Zhou Y, Zhang H, Li W, Niu B, Chen J. A mechanically adaptive “all-sugar” hydrogel for cell-laden injection. *European Polymer Journal* 2022;174:111328.
